# Autophagy Activation Is Involved in Acidic Fibroblast Growth Factor Ameliorating Parkinson’s Disease *via* Regulating Tribbles Homologue 3

**DOI:** 10.3389/fphar.2019.01428

**Published:** 2019-12-02

**Authors:** Xingfeng Zhong, Beini Wang, Guanyinsheng Zhang, Yuan Yuan, Xiaoli Hu, Jun Xiong, Peipei Zheng, Yaqian Liu, Ke Xu, Jian Xiao, Yanqing Wu, Junming Ye

**Affiliations:** ^1^Department of Anesthesia, The First Affiliated Hospital, Gannan Medical University, Ganzhou, China; ^2^Department of Anesthesia, Affiliated Hospital of Guizhou Medical University, Guiyang, China; ^3^Molecular Pharmacology Research Center, School of Pharmaceutical Science, Wenzhou Medical University, Wenzhou, China; ^4^The Institute of Life Sciences, Wenzhou University, Wenzhou, China

**Keywords:** Parkinson’s disease, acidic fibroblast growth factor, autophagy, endoplasmic reticulum stress, tribbles homologue 3

## Abstract

Parkinson’s disease (PD) is a degenerative disorder of the central nervous system, resulting in loss of dopamine neurons. Excessive endoplasmic reticulum (ER) stress and autophagy dysfunction play a crucial role on Parkinson’s disease (PD) development. It has been showed that acidic fibroblast growth factor (aFGF) alleviates the development of PD by inhibiting ER stress. But the role of autophagy and its relationship with ER stress during aFGF treatment for PD has not been elucidated. We found that both aFGF and rapamycin (Rapa) improved 6-Hydroxy Dopamine (6-OHDA)-induced PD development as shown with histomorphology results in striatum and substantia nigra (SNpc). Additionally, aFGF promoted autophagy with increasing mTOR and decreasing p62 expressions, and then exerts its neuroprotective role in 6-OHDA-treated PC12 cells, which were abolished by chloroquine (CQ) treatment. Moreover, 4-phenylbutyric acid (4-PBA) administration inhibited the expressions of autophagy markers during 6-OHDA-treated PC12 cells, which was similar with aFGF treating PC12 cells under 6-OHDA condition. Furthermore, we had detected the expressions of CHOP and its downstream factor, tribbles homologue 3 (TRB3), a pro-apoptotic protein. We found that TRB3 and CHOP expressions were significantly downregulated after treating with aFGF and 4-PBA in 6-OHDA-treated PC12 cells and PD model. Taken together, this study has demonstrated that aFGF treatment ameliorates 6-OHDA-induced elevated ER stress and subsequently suppression of autophagy *via* inhibiting TRB3 activation, and consequently ameliorates 6-OHDA-induced neurotoxicity.

## Introduction

Parkinson’s disease (PD) is a kind of neurodegenerative disease, which is associated with the abnormally accumulated α-synuclein (α-syn) in the substantia nigra pars compacta (SNpc). In the early study of PD development, gene mutation of α-syn on the autosome caused excessive aggregation damage (Fujiwara et al., 2002; Hasegawa et al., 2002; Samuel et al., 2016; Longo et al., 2017). The cellular mechanisms, include autophagy (Magalhaes et al., 2016), endoplasmic reticulum (ER) stress (Kuang et al., 2010; Ning et al., 2019), oxidative stress (Ding et al., 2018; Mamelak, 2018), and others, are involved in the amelioration of misfolded protein. Thus, regulation of cellular stress may be the target for treating PD.

Acidic fibroblast growth factor (aFGF or FGF1) is an important member of fibroblast growth factors, which can be synthesized and released from neuron (Walicke and Baird, 1988). aFGF exerts neuroprotective role in the peripheral and central nervous systems (Tsai et al., 2015; Wu et al., 2017; Aiki et al., 2018; Ko et al., 2018). The previous studies have showed that aFGF can attenuate 6-OHDA-induced dopaminergic neuron toxicity (Wei et al., 2014). In addition, aFGF protects human primary neurons against gp120 toxicity, and overexpression of aFGF alleviates the neurodegeneration of gp120 mice (Everall et al., 2001; Crews et al., 2009). The mechanism underlying aFGF treating for PD is through inhibiting ER stress (Wei et al., 2014). Promotion of autophagy is the mechanism during aFGF treating for spinal cord injury (Li et al., 2018). However, there is no studies to reveal on role of autophagy, and its’ relationship with ER stress during aFGF treating for PD.

Autophagy is an adaptive process in body, which degrades and recycles the abnormal cytoplasmic components and organelles to provide nutrients for cells. Hyperactivation of autophagy triggers cell death program. In general, autophagy regulates cell fate depending on cell type, specific environment and predisposing factors (Jin et al., 2012). Accumulating evidences suggest that autophagy is impaired in PD (Lee et al., 2015). Under normal condition, a small amount of aggregated α-syn will be degraded by the giant autophagy pathway (Cao et al., 2017), however, the mutantion of α-syn can inhibit the degradation process of autophagy (Song et al., 2014; Winslow and Rubinsztein, 2014; Lohmann et al., 2019). Previous studies have also found that the enhancement of autophagy can alleviate the toxic effect of α-syn mutation on midbrain dopaminergic neurons (Decressac et al., 2013). These studies indicate that promotion of autophagy is very important for PD treatment.

The endoplasmic reticulum (ER) is responsible for the synthesis and assembly of protein in cell. Once the misfolded protein is accumulated, it triggers an unfolded protein response (UPR), but an excessively sustained response to decompensation leads to the appearance of ER stress, which in turn causes cell death. Recent studies have shown that inhibition of ER stress protects dopaminergic neurons (Shan et al., 2019). Neuronal apoptotic kinase, known as tribbles homologue 3 (TRB3), is a novel protein that induces cell death by ER stress, which is also related to the regulation of autophagy. Normally, the expression of TRB3 in brain is extremely low. Once stress is triggered, TRB3 is sharply increased. Moreover, the expression of TRB3 is significantly increased during development of PD (Aim et al., 2015) and AD (Saleem and Biswas, 2017). However, it is unclear whether aFGF alleviates PD development by suppressing TRB3 expression.

Multiple studies have also shown a crosstalk between ER stress and autophagy (Li et al., 2017; Yin et al., 2017; Zhou et al., 2017). When ER stress occurs, autophagy can be triggered by unfolded protein reactions (UPR) to remove excessive unfolded proteins and damaged organelles, and maintain cell homeostasis. Moreover, TRB3 plays a key bridge connection between ER stress and autophagy (Salazar et al., 2009; Aim et al., 2015; Tang et al., 2015; Ord and Ord, 2017). Therefore, we have used 6-OHDA to induce PD model or PC12 cells model *in vivo* and *in vitro*, and investigated the role of autophagy regulation during aFGF treating for PD *via* treating with aFGF, rapamycin (Rapa)-autophagy inducer and chloroquine (CQ)-autophagy inhibitor. Moreover, 4-phenylbutyrate (4-PBA)-ER stress inhibitor is administrated to investigate the relationship between ER stress and autophagy during these processes.

## Materials and Methods

### Animals and Surgical Procedures

One hundred ten adult male SD rats (220–250 g) were purchased from the Animal Center of the Chinese Academy of Science (Shanghai, China). The rats were housed under a 12-h light/dark cycle at 21–23°C and provided access to food and water *ad libitum*. All procedures were approved by Laboratory Animal Ethics Committee of Wenzhou Medical University. After anesthetizing with 10% chloral hydrate, the head of animal was shaved and sterilized. Then, the rat was fixed on the rat brain stereogram (KOPF, Germany), and the 5% compound lidocaine cream was applied to the front of head. After the drug was absorbed, the longitudinal cleavage revealed the fascia, followed by 3% hydrogen peroxide exposing the front point on the skull. The 6-OHDA (10 µl, 2 µg/ul dissolved in 0.2% ascorbic acid, Sigma-Aldrich, St. Louis, MO, USA) was injected into the dental drill hole at the right side of position the striatum (coordinates: A: anterior +0.7 mm, L: 2.8 mm from the midline, H: +5.5 mm) using micro syringe. The injection speed was 10 nl/s, and the needle was left for 10 min after injection. The sham-operation model was performed same surgical procedures and injected an equal volume of 0.2% ascorbic acid.

### Behavioral Test

One week after the right striatum stereotactic injection of 6-OHDA, the animals were tested for rotational behavior. Rats were intraperitoneally injected with apomorphine hydrochloride (0.5 mg/kg), as a dopamine receptor agonist, the dopamine receptor on the injured side is up-regulated, which can cause the rotation of the rats in Parkinson’s disease to the healthy side. The device was used to detect the rotational behavior of the rats. The number of rotations of the rats to the opposite side of the injury was recorded within 30 min. The successful PD model rotated to the left side more than 7 turns/min. Then, 72 successful Parkinson’s disease rats were randomly divided into six groups (n = 12): PBS, aFGF (80 µg/kg/day, tail vein), CQ (50 mg/kg/day, intraperitoneally), Rapa (1.5 mg/kg/day, intraperitoneally), aFGF + CQ, and 4-PBA (100 mg/kg/day, intraperitoneally). In addition, the sham-operation group (n = 12) was injected with PBS in the tail vein for 2 weeks. The rotational behavior test of rats was performed at 1, 2 and 3 weeks after surgical procedure and treatment.

### Cell Culture and Treatment

PC12 cells were purchased from the Cell Storage Center of Wuhan University (Wuhan, China). PC12 cells were cultured in Dulbecco’s Modified Eagle Medium (DMEM, Invitrogen, Carlsbad, CA) supplemented with 10% fetal bovine serum (FBS, Invitrogen) and 1% antibiotics (100 units/ml penicillin, 100 µg/ml streptomycin). They were incubated in a humidified atmosphere containing 5% CO_2_ at 37 °C. The cells were seeded in a 96-well plate at density of 1 × 10^4^, then treated with 6-OHDA at different doses of 25 µM, 50 µM, 75 µM, 100 µM, 125 µM, 150 µM, 175 µM and 200 µM for 24 h. Then, the cells were incubated with CCK-8 assay (Cell counting kit-8) at 37°C for 2 h, and measured the optical density at 450 nm. The experiments were divided into six groups: PBS, aFGF (20 ng/ml), CQ (75 µM), aFGF + CQ, Rapa (1 µM), and 4-PBA (1 mM).

### Hematoxylin and Eosin (H&E) Staining and Nissl Staining

After anesthesia, the brain was dissected immediately and placed on the ice, then fixed 4% with paraformaldehyde (PFA) for 24 h in 4 °C. Subsequently, paraffin-embedded brain regions containing substantia nigra and striatum were sliced at 4 um thickness. For Nissl staining, after dewaxed and hydrated, tissue sections were stained with cresol violet and Nissl differentiation solutions according to the instructions (Beyotime) and acquired the image using light microscopy. For H&E staining, the sections were stained with hematoxylin and eosin reagent, and observed under light microscope.

### Immunohistochemical Staining

After anesthesia, the brain was dissected immediately and placed on the ice, then fixed 4% with paraformaldehyde (PFA) for 24 h in 4°C. Subsequently, paraffin-embedded brain regions containing substantia nigra and striatum were sliced at 4 um thickness. After dewaxed and hydrated, the sections were incubated with 3% hydrogen peroxide for 15 min, and antigen retrieval at high temperature and pressure. Then, after blocking with 5% bovine serum albumin (BSA) for 30 min at 37 °C, the sections were incubated overnight at 4 °C with the following primary antibodies: TH (1:500, Abcam), α-syn (1:250, Millipore), CHOP (1:100, Proteintech), TRB3 (1:50, Santa Cruz Biotechnology), mTOR (1:250, Cell Signaling Technology), p62 (1:250, Abcam). Then, the sections were washed three times with PBST and incubated the horseradish peroxidase-conjugated secondary antibody at 37 °C for 2 h. Finally, these sections were reacted with 3,3-diaminobenzidine (DAB, 1:20 dilution) for 5–10 min. Hematoxylin was used to stain the nucleus. The sham operation group was used as a negative control. The signals were analyzed by counting the number of positive cells in region of interest at 400× of microscope of Nikon ECLPSE 80i (Nikon, Tokyo, Japan). The optical density of TH, α-syn, CHOP, TRB3, mTOR and p62 was randomly selected from five samples in each sample.

### Immunofluorescence Staining

The cells were inoculated on coverslips, and then fixed with 4% PFA for 25 min at 4 °C. Subsequently, the cells were washed in PBS three times. The cells were incubated with 5% BSA in PBS containing 0.1% Triton X-100 for 30 min at room temperature. Then, the sections were incubated at 4°C overnight with the following primary antibodies: CHOP (1:50, Proteintech), TRB3 (1:50, Santa Cruz Biotechnology), mTOR (1:250, Cell Signaling Technology), and p62 (1:250, Abcam). After washing with PBS three times for 5 min, the sections were incubated with AlexaFluor-488 or AlexaFluor-647 donkey anti-rabbit/mouse secondary antibodies for 1 h at room temperature. After washed with PBS, the sections were staining the nuclei with DAPI and captured the image on a confocal fluorescence microscope (Nikon, A1 PLUS, Tokyo, Japan).

### Tunel Staining

TUNEL staining was performed using the ApopTag Fluorescein Direct *In Situ* Apoptosis Detection Kit (Roche, Basel, Switzerland). According to the standard protocol, the fixed cell slides were incubated with 20 µg/ml proteinase K solution for 10 min at room temperature. The slides were then rinsed with PBS three times, which was followed by incubation with the TUNEL reaction mixture for 1 h at 37 °C. After rinsing with PBS three times for 5 min, sections were treated with 4 ’, 6-diamidino-2-pheny-lindole (DAPI, Beyotime, Shanghai, China) for 5 min at room temperature and mounted with aqueous mounting medium. The results were imaged using a Nikon ECLIPSE 80i microscope (Nikon, Tokyo, Japan). Quantification was performed by counting the TUNEL-positive cells number in five random fields using ImageJ software.

### Western Blot Analysis

The midbrain and PC12 cells were collected and lysed using RIPA buffer (20 mM Tris-HCl, pH7.5, 150 mM NaCl, 1 mM EDTA, 1% Triton-X100, 0.5% sodium dexoycholate, 1 mM PMSF and 10 µg/ml leupeptin). The lysate was centrifuged at 12,000*g* for 20 min at 4°C, and the supernatant was quantified with BCA reagents (Thermo, Rockford, IL, USA). Proteins (30 µg) were separated on a 12% gel and transferred onto a 0.22 µm PVDF membrane (Bio-Rad, Hercules, CA, USA). The membrane was blocked with 5% (w/v) non-fat milk (Bio-Rad) in Tris-buffered saline with 0.1% Tween-20 (TBST) for 0.5 h at room temperature, and then the membranes were incubated overnight at 4°C with the following primary antibodies: TH (1:1000, Abcam), α-syn (1:1,000, Abcam), FGFR1 (1:1,000, CST), GRP78 (1:1,000, Abcam), CHOP (1:500, Proteintech), TRB3 (1:100, Santa Cruz Biotechnology), LC3A/B (1:1,000, CST), mTOR (1:1,000, CST), p-mTOR (1:1,000, CST), and p62 (1:1,000, CST). The membranes were washed with TBST three times and incubated with horseradish peroxidase-conjugated secondary antibodies for 2 h at room temperature. Then, the signals were detected using the Chemi DocXRS + Imaging System (Bio-Rad), and the bands were quantified using densitometric measurement by the Quantity-One software. All experiments were repeated three times.

### Statistical Analysis

Data were presented as mean ± SEM from three individual experiments. All cells culture experiments were conducted in triplicate. Statistical analysis and mapping were performed using GraphPad Prism 5. Except for rat animal behavior using repeated measures analysis of variance, one-way analysis of variance (ANOVA) followed by Tukey’s *post hoc* test were used for analyzing significant difference. Multiple comparisons were used to compare between groups. *P <0.05* was used to indicate significant differences in data.

## Results

### aFGF Treatment Ameliorates 6-OHDA-Induced PD Disease *via* Activating Autophagy

**Figure 1:**
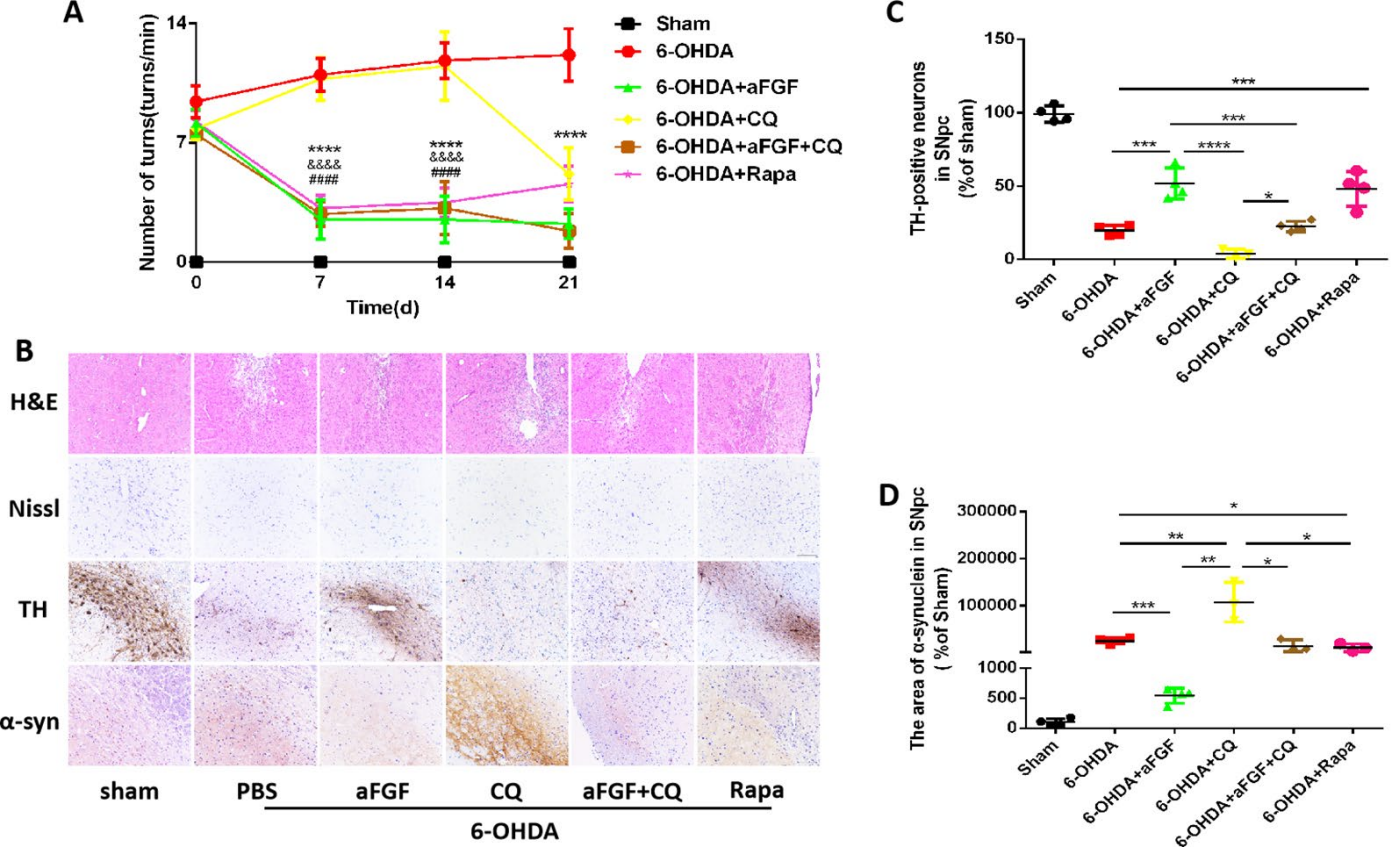
Effect of autophagy on aFGF attenuating 6-OHDA-induced PD. **(A)** The number of rotations (turns/min) on the affected side of rats during rotational behavior test, n = 12. ****P <0.0001: aFGF group *vs.* 6-OHDA group; *^####^P < 0.0001*: aFGF group *vs.* CQ group; ^&&&&^ *P < 0.0001*: aFGF + CQ group *vs.* CQ group. **(B)** H&E staining of striatum region, Nissl staining of nigral region, and immunohistochemistry staining results of TH and α-syn expression in striatum region. Scale bar = 50 μm, n = 6. **(C**, **D)** Quantitative analysis of immunohistochemistry staining results of TH and α-syn expression in striatum region, *P < 0.05, **P < 0.01, ***P < 0.001, ****P < 0.0001 vs. the other group, n = 6.

To evaluate the effect of autophagy in aFGF ameliorating 6-OHDA-induced PD, we measured the rotational behavior of APO-induced rat at 7, 14 and 21 days after administration with PBS, aFGF, CQ, aFGF+CQ, and Rapa. The results of rotational behavior showed that from day 0 to 21, the number of turns in 6-OHDA group did not significantly decrease, which is similar with that in CQ group. Compared with that in 6-OHDA group, aFGF treatment significantly decreased the number of turns of 6-OHDA mice, indicating aFGF effectively alleviates the rotational behavior of PD rats. Additionally, the behaviors of PD rats from aFGF + CQ group and Rapa group have significant improved when compared with that in CQ group (**Figure 1A**). These results demonstrated that aFGF may ameliorate PD *via* enhancing autophagy.

We also assessed the striatum morphology and the number of Nissl bodies in nigral to confirm this hypothesis. It was found that the rats in 6-OHDA group and CQ group have showed damaged striatum, irregular hyperplasia and scar, decreased Nissl bodies, which were reversed by aFGF or Rapa treatment. Moreover, compared with CQ group, aFGF + CQ treatment significantly relieved the damage of 6-OHDA on striatum morphology and Nissl bodies (**Figure 1B**), indicating that aFGF treatment can block the inhibition of autophagy and ameliorate the striatum injury and death of SNpc neurons in PD. Furthermore, we had detected the TH and α-syn expressions in the SNpc of rats by immunohistochemical staining (**Figure 1B**). It was observed that the rats from 6-OHDA and CQ group have shown few TH-positive neurons and abundant α-syn deposition in the SNpc, and aFGF and Rapa treatment remarkably block them (**Figures 1C, D**). More importantly, aFGF + CQ treatment increased the TH level and reduced α-syn deposition in the substantia nigra region, but its effect was worse than that in aFGF treatment. Taken together, these indicate that aFGF may enhance TH level and decrease α-syn deposition in the substantia nigra neurons of PD rats by activating autophagy, and finally achieve its neuroprotective effect.

### aFGF Treatment Attenuates 6-OHDA-induced PD *via* Inhibiting ER Stress

**Figure 2:**
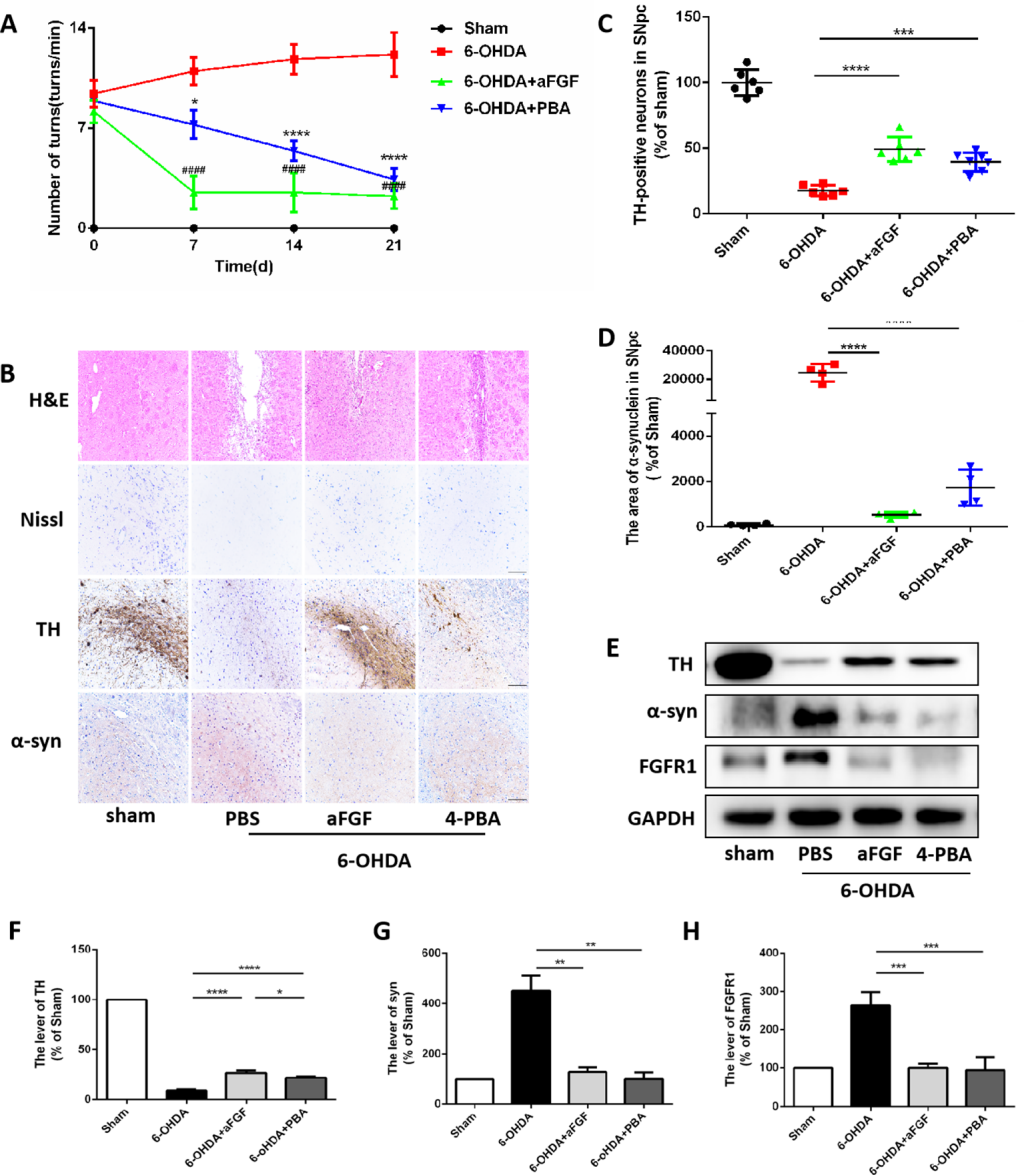
aFGF treatment attenuates 6-OHDA-induced PD via inhibiting ER stress. **(A)** The number of rotations (turns/min) on the affected side of rats during rotational behavior test, n = 12. *^####^* *P < 0.0001*: aFGF group *vs.* 6-OHDA group; ****P < 0.0001: 4-PBA group *vs.* 6-OHDA group. **(B)** H&E staining of striatum region, Nissl staining of nigral region, and immunohistochemistry staining results of TH and α-syn expression in striatum region. Scale bar = 50 μm, n = 6. **(C**, **D)** Quantitative analysis of immunohistochemistry staining results of TH and α-syn expression in striatum region. **(E)** Western blot results of TH, α-syn and FGFR1 expressions. **(F**–**H)** Quantitative analysis results of TH, α-syn, and FGFR1 expressions, *P < 0.05, **P < 0.01, ***P < 0.001, ****P < 0.0001 vs. the other group, n = 6.

We have further confirmed the effect of ER stress on aFGF ameliorating 6-OHDA-induced PD. As shown in **Figure 2A**, aFGF and 4-PBA treatment significantly decreased the number of turns of PD rat, indicating that aFGF may improve the symptoms of PD by inhibiting ER stress. Then, we found that the rats in 6-OHDA group have showed damaged striatum, irregular hyperplasia and scar, decreased Nissl bodies, which are reversed by aFGF or 4-PBA treatment (**Figure 2B**). Moreover, aFGF and 4-PBA treatment blocked 6-OHDA-induced loss of TH-positive neurons and α-syn deposition in the substantia nigra neurons (**Figures 2B–D**). Consistent with the results of immunohistochemical staining, western blot results showed that 4-PBA and aFGF administration increases TH expression and promotes α-syn degradation, and ameliorates 6-OHDA-induced increase FGFR1 expression (**Figures 2E–H**).

### aFGF Administration Activates Autophagy Level by Inhibiting ER Stress During PD Development

**Figure 3:**
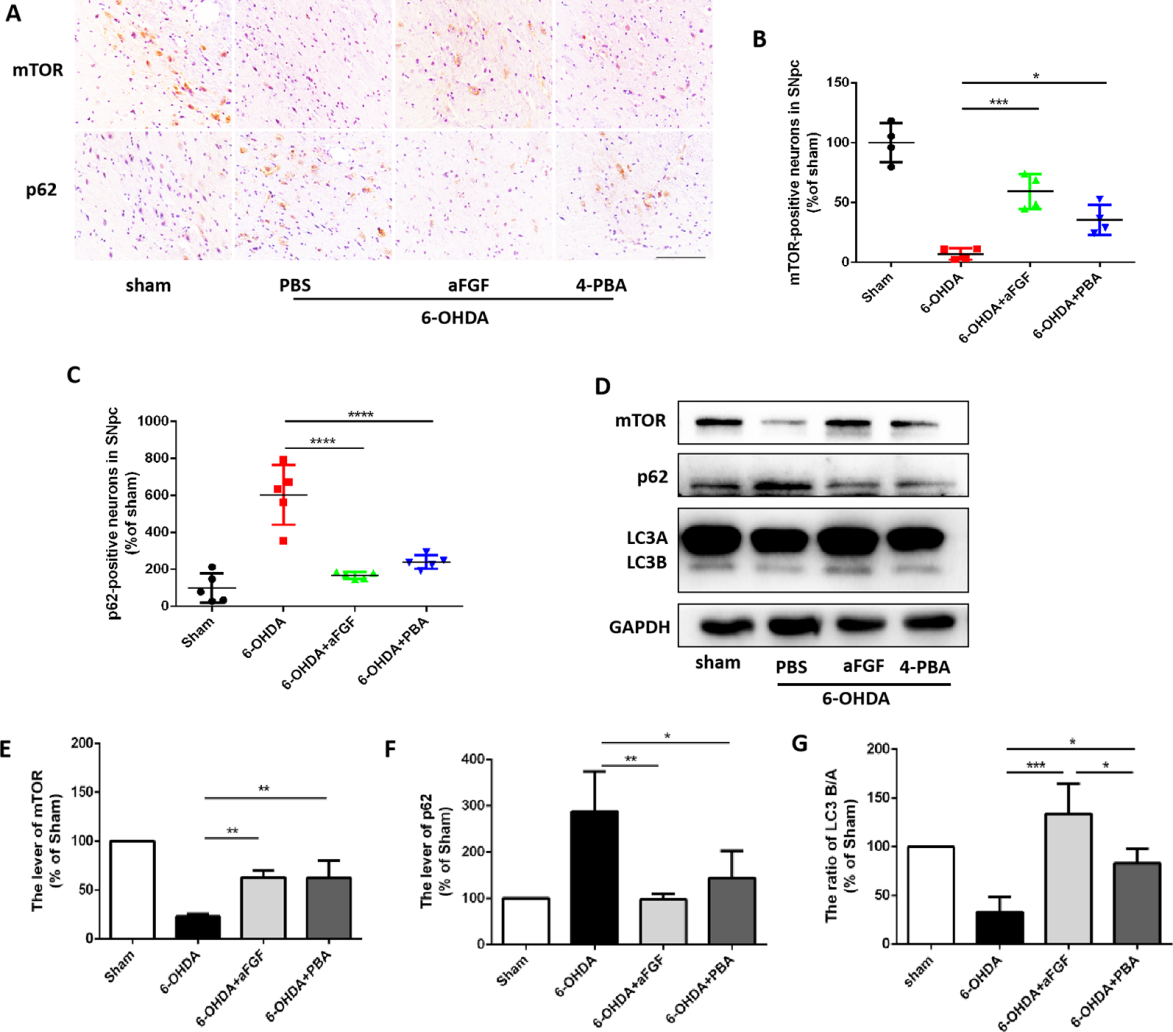
aFGF administration activates autophagy level by inhibiting ER stress during PD treatment. **(A**–**C)** Immunohistochemical image and quantitative analysis of mTOR and p62 protein expressions in the substantia nigra of the affected side. Scale bar = 50 μm, n = 6. **(D)** Western blot results of mTOR, p62 and LC3 expressions. **(E**–**G)** Quantitative analysis of mTOR, p62 and LC3 expressions. *P < 0.05, **P < 0.01, ***P < 0.001, ****P<0.0001 vs. the other group, n = 6.

Based on our prior study, it was found that ER stress and autophagy are involved in neuroprotective role of aFGF during PD treatment. Next, we have further detected the relationship of ER stress and autophaghy during aFGF treating with PD. Immunohistochemistry results showed that both 4-PBA and aFGF treatement promote autophagic flow by inhibiting mTOR activity and promoting p62 degradation (**Figures 3A–C**). Furthermore, western blot results showed that both 4-PBA and aFGF administration inhibit mTOR activity, increase LC3B expression and promote p62 degradation (**Figures 3D–G**). All of these studies suggest that aFGF promotes autophagy level and exerts its neuroprotective role during PD *via* suppressing ER stress.

### aFGF Suppresses the Pro-Apoptotic Protein of TRB3 and Subsequently Activates Autophagy *via* Inhibiting CHOP Expression During PD Treatment

**Figure 4:**
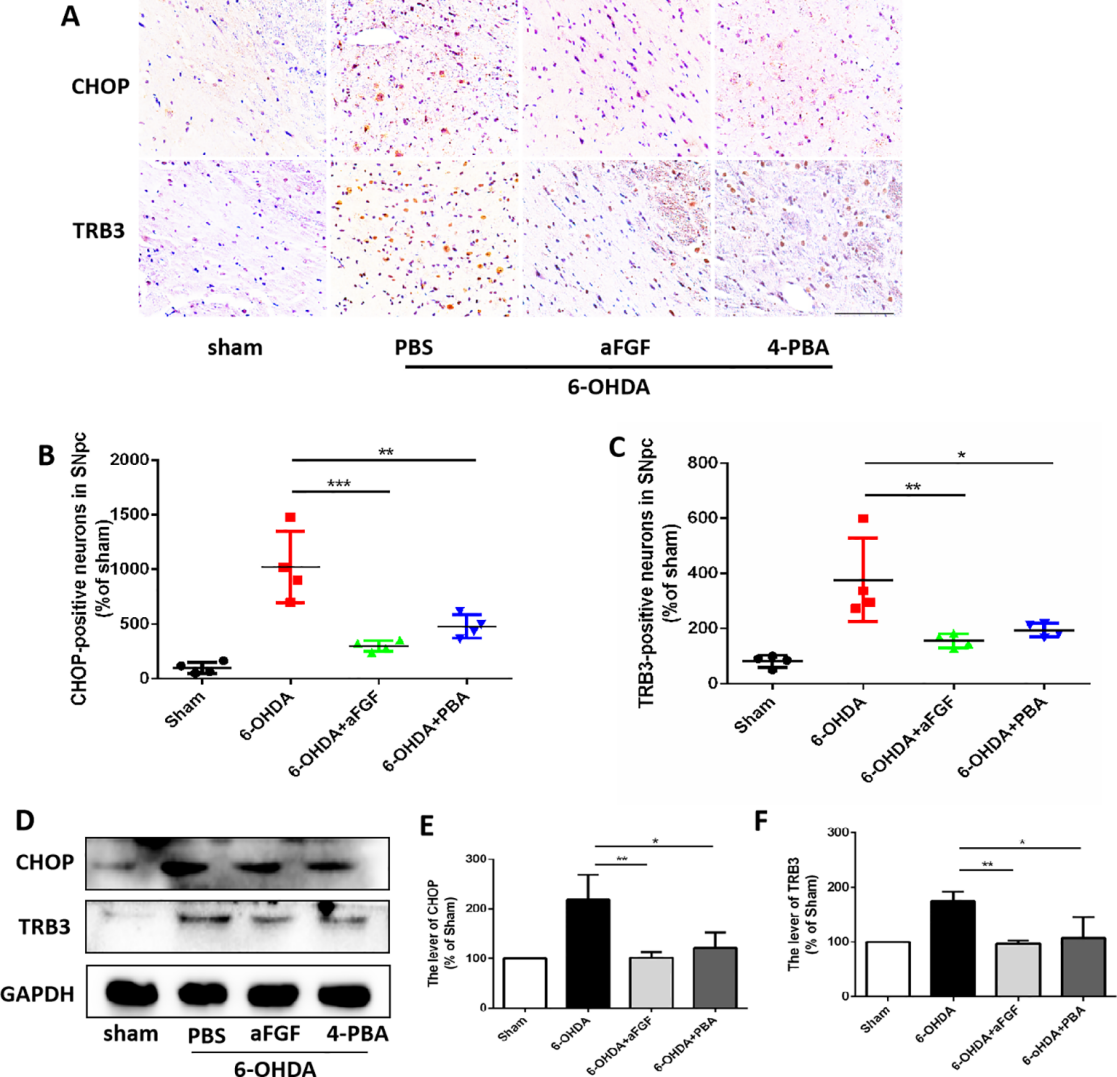
aFGF suppresses the pro-apoptotic protein of TRB3 and subsequently activates autophagy via inhibiting CHOP expression during PD development. **(A**–**C)** Immunohistochemical image and quantitative analysis of CHOP and TRB3 expressions in the substantia nigra of the affected side. Scale bar = 50 μm, n = 6. **(D)** Western blot results of CHOP and TRB3 expressions. **(E**, **F)** Quantitative analysis of CHOP and TRB3 expressions. *P < 0.05, **P < 0.01, ***P < 0.001 vs. the other group, n = 6.

Progressive loss of DAergic is the main pathological manifestation of PD, and previous studies have shown that aFGF can protect neurons by inhibiting ER stress. It is reported that TRB3 is a protein that involved in ER stress-associated apoptosis and autophagic cell death. We speculated that TRB3 is critical factor for ER stress related with autophagy during aFGF treating for PD. Using immunohistochemical staining and western blot assay (**Figures 4A–F**), it was observed that 6-OHDA treatment significantly induces the expression levels of CHOP and TRB3, and both aFGF and 4-PBA treatment reduce the overexpression of these two proteins. Taken together, these studies indicate that aFGF suppresses the pro-apoptotic protein TRB3 and subsequently activates autophagy *via* inhibiting CHOP expression during PD development.

### Effect of Autophagy on aFGF Attenuating 6-OHDA-Induced Apoptosis in PC12 Cells

**Figure 5:**
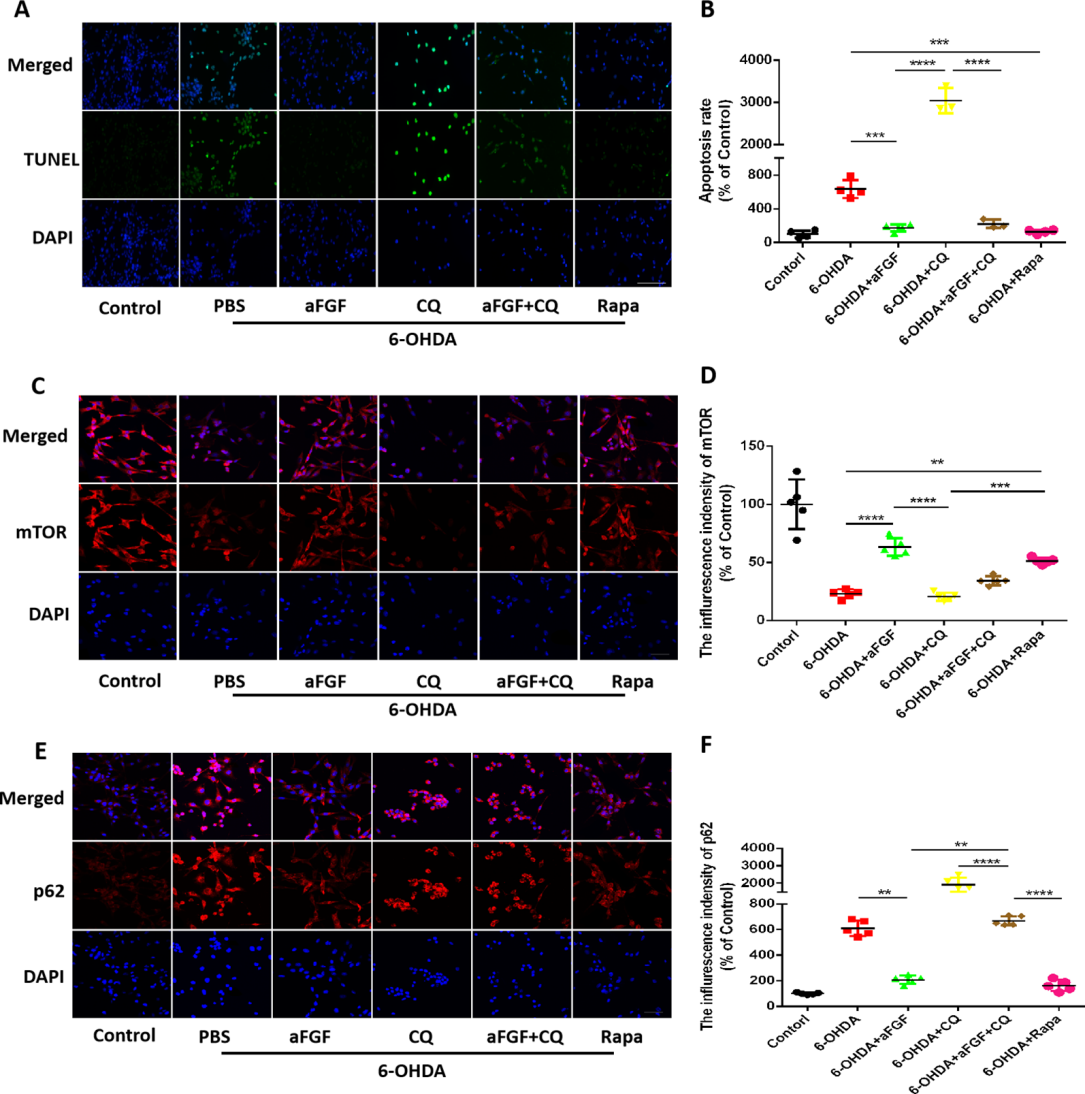
Effect of autophagy on aFGF attenuating 6-OHDA-induced apoptosis in PC12 cells. **(A)** Representative images of TUNEL staining (green) of PC12 cells under 6-OHDA treatment; **(B)** Quantitative analysis of TUNEL-positive cells. Scale bar = 50 μm, n = 5. **(C**, **D)** Representative fluorescence images and quantitative analysis of mTOR expression in PC12 cells. Scale bar = 50 μm. **(E**, **F)** Representative fluorescence images and quantitative analysis of p62 expression in PC12 cells. Scale bar = 50 μm. **P < 0.01, ***P < 0.001, ****P < 0.0001 vs. the other group, n = 5.

Here, we had further investigated whether aFGF can attenuate neuronal apoptosis by regulating autophagy *in vitro*. As is shown in **Figures 5A, B**, aFGF and Rapa treatment significantly reduced 6-OHDA-triggered apoptotic cells. In addition, TUNEL-positive cells in CQ group was significantly increased compared with that in 6-OHDA group, while the number of apoptotic cells in aFGF + CQ group was significantly lower than that in CQ group (**Figures 5A, B**). These data suggest that aFGF alleviates neuronal apoptosis by regulating autophagy under 6-OHDA treatment. Then, we had further detected the expressions of autophagy related protein, and found that 6-OHDA or CQ treatment and significantly decreases the expression of mTOR and increases p62 expression, which are reversed by aFGF and Rapa treatment (**Figures 5C–F**). Moreover, there is significant difference of the expressions of mTOR and p62 between the aFGF + CQ and CQ group (**Figures 5C–F**). All of these studies indicate that aFGF promotes autophagic circulation and ameliorates 6-OHDA-induced neuronal apoptosis *in vitro*.

### aFGF Administration Activates Autophagy and Protects Neuron by Inhibiting ER Stress-TRB3 Signaling *In Vitro*

**Figure 6:**
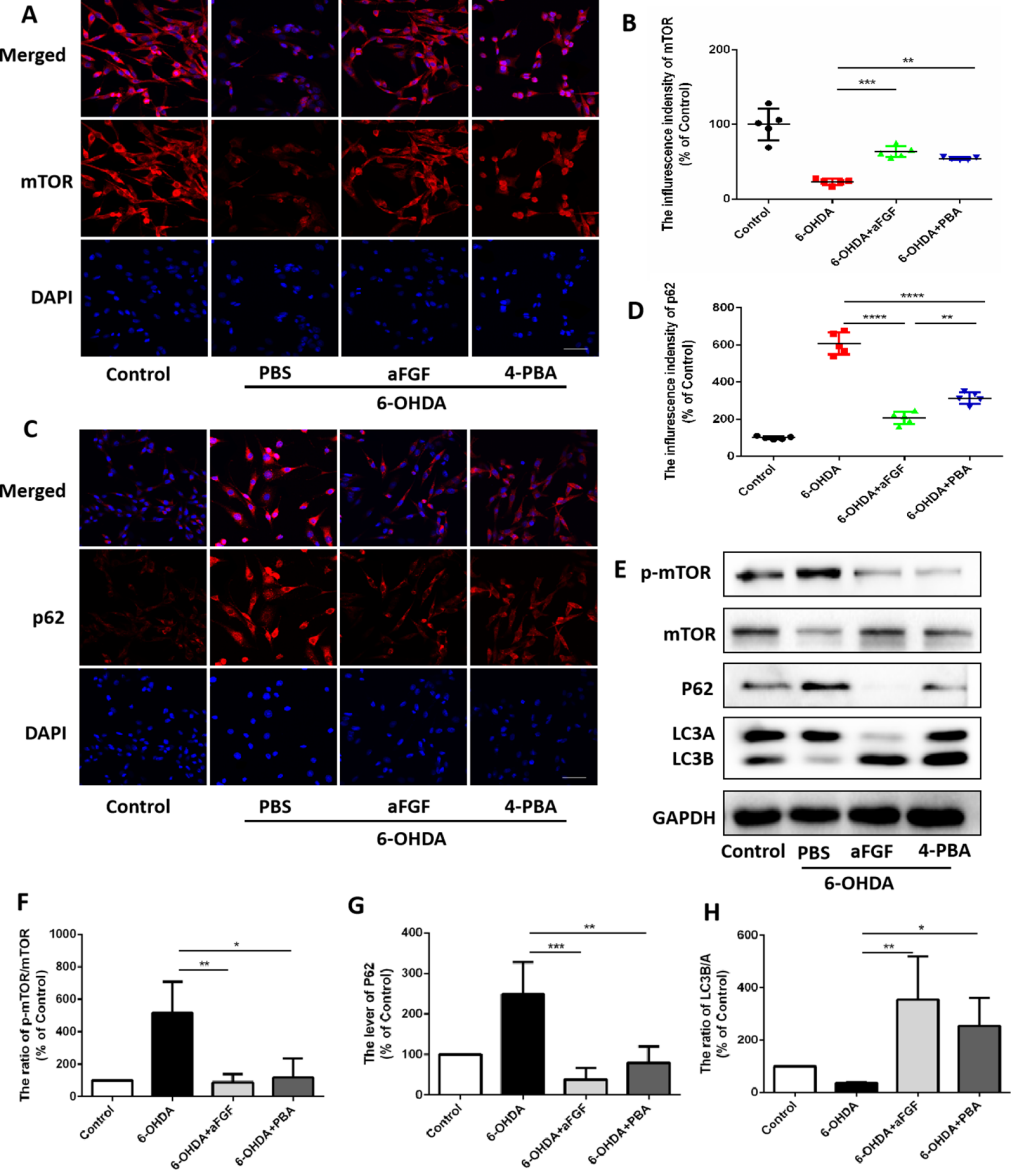
aFGF administration activates autophagy by inhibiting ER stress in PC12 cells. **(A**–**D)** Representative fluorescence images and quantitative analysis of mTOR and p62 expressions in PC12 cells. Scale bar = 50 μm, n = 5. **(E)** Western blot results of p-mTOR, mTOR, p62 and LC3 expressions in PC12 cells. **(F**–**H)** Quantitative analysis results of p-mTOR, mTOR, p62 and LC3 expressions in PC12 cells. *P < 0.05, **P < 0.01, ***P < 0.001, ****P < 0.0001 vs. the other group, n = 3.

Here, we had further assessed the relationship of ER stress and autophaghy during aFGF treating PC12 cells under 6-OHDA condition. Consistent with the results *in vivo*, 6-OHDA treatment significantly decreased the expressions of mTOR and LCB, and increased p62 expression in PC12 cells, and both aFGF and 4-PBA treatment remarkably blocked them (**Figures 6A–H**), indicating that aFGF activated autophagy by inhibiting ER stress in PC12 cells

**Figure 7:**
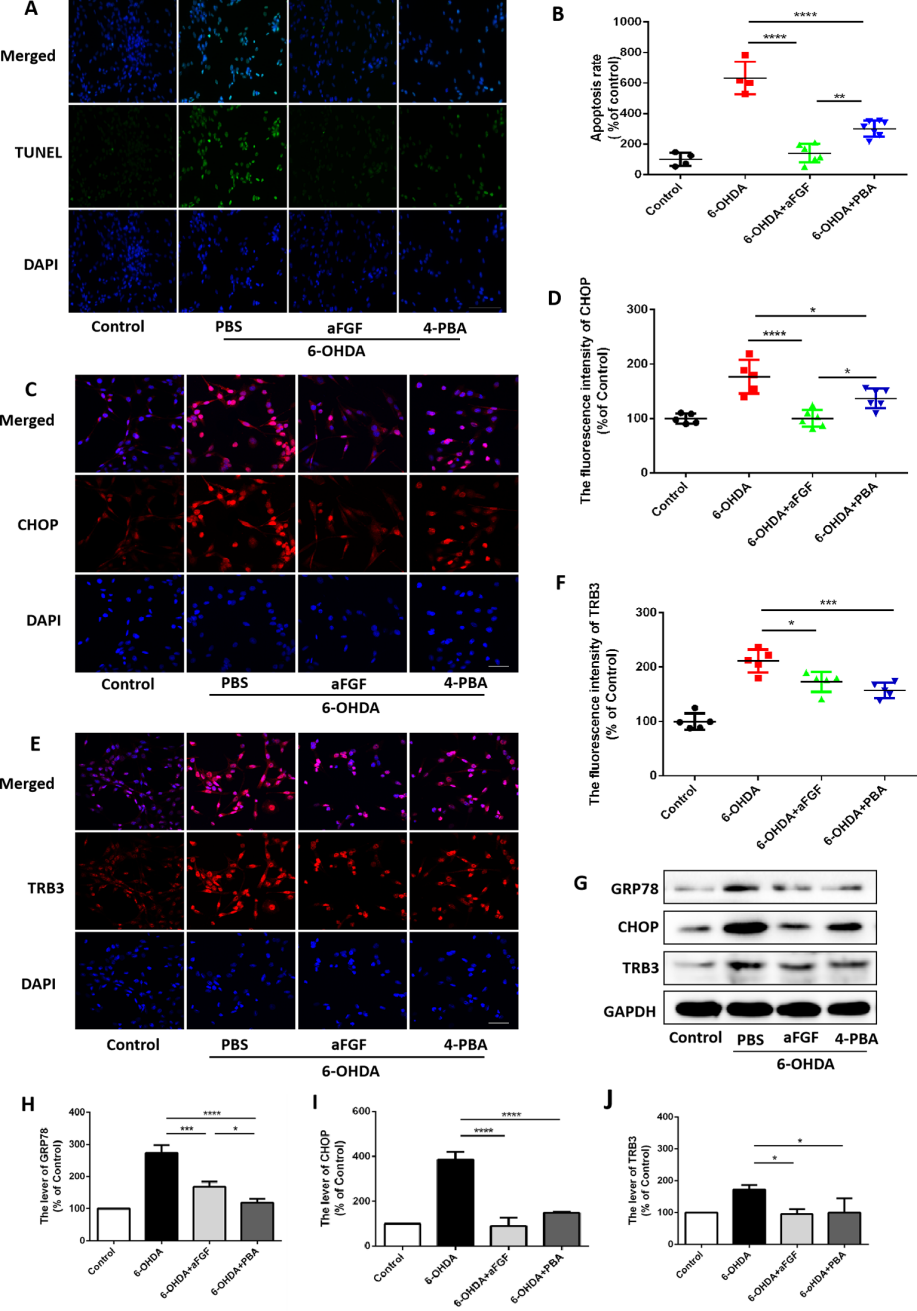
aFGF treatment ameliorates 6-OHDA-induced neuronal death via suppressing TRB3 expression. **(A)** Representative images of TUNEL staining (green) of PC12 cells under 6-OHDA treatment. **(B)** Quantitative analysis of TUNEL-positive cells, Scale bar = 50 μm, n = 5. **(C**–**F)** Representative fluorescence images and quantitative analysis of CHOP and TRB3 expressions in PC12 cells. Scale bar = 50 μm, n = 5. **(G)** Western blot results of GRP78, CHOP, and TRB3 expressions in PC12 cells. **(H**–**J)** Quantitative analysis results of GRP78, CHOP, and TRB3 expressions in PC12 cells, n = 3. *P < 0.05, **P < 0.01, ***P < 0.001, ****P < 0.0001 vs. the other group.

Then, we had investigated whether inhibition of ER stress-TRB3 signaling is involved in aFGF ameliorating 6-OHDA-induced neuronal apoptosis. TUNEL staining results showed that the numbers of TUNEL-positive PC12 cells in aFGF and 4-PBA treated-group are significantly reduced when compared with that in 6-OHDA group (**Figures 7A and B**), indicating that aFGF exerts its neuroprotective role by inhibiting ER stress. Then, using immunofluorescence staining and western blot, we detected the expressions of GRP78, CHOP and TRB3 in PC12 cells, and found that both aFGF and 4-PBA treatment decrease 6-OHDA-induced increases of GRP78, CHOP and TRB3 expressions (**Figures 7C–J**). These studies confirm that aFGF administration activates autophagy and exerts its neuroprotective role by inhibiting ER stress-TRB3 signaling *in vitro*.

## Discussion

With the rapid growth of the elderly population, the incidence of PD is also increasing. However, the effective treatments for PD are still limited. At present, there are many studies focused on investigating the neuroprotective drugs, and neurotrophic factors have attracted much attention in recent years. aFGF, a important member of FGFs, has been reported to have neuroprotective effect (Tsai et al., 2015; Wu et al., 2017). Increased expression of aFGF was observed during central neuronal injury (Walicke and Baird, 1988), which indirectly indicates that aFGF is important for neuronal survival. Previous studies have shown that aFGF has a protective effect on 6-OHDA-induced DAergic injury (Wei et al., 2014). As a disease associated with excessive accumulation of misfolded proteins, α-syn accumulation promotes PD progression. ER and autophagy are essential for the normal protein synthesis and translocation in body. Our current study has shown that aFGF ameliorates the development of PD by regulating autophagy and ER stress. The specific mechanism may be related to aFGF down-regulating TRB3 *via* inhibiting ER stress, and then activating autophagy to protect DAergic from 6-OHDA.

6-OHDA is a neurotoxin that selectively damages DAergic in SNpc. A single injection of 6-OHDA into striatum can be used to simulate the chronic progression of classical PD model. Autophagy is generally an adaptive mechanism that allows for the orderly degradation and recycling of cellular components. It has shown that the autophagy level is significantly decreased in serious of degenerative diseases (Pickford et al., 2008; Spencer et al., 2009; Menzies and Rubinsztein, 2010), indicating that promotion of autophagy may protect DAergic during PD development (Darabi et al., 2018). In our current study, a rat model of PD was established using 6-OHDA induction and behavioral evaluation indicators. According to behavioral indicators, it was found that on the 7th and 14th days after administration, the behavior of PD rat was significantly improved after treating with aFGF and Rapa, while CQ treatment not only did have no remission, but also promoted the neuronal death of PD rats, which is due to the inhibition of autophagy level in PD (Michiorri et al., 2010). In addition, morphological staining results are consistent with that in behavioral test. However, the CQ group behavior was significantly improved after CQ with drawal for 1 week, which may be related to drug half-life. Tyrosine hydroxylase (TH) is a maker protein in DAergic. Immunohistochemical staining results have shown that both aFGF and autophagy activatorcould alleviate the loss of TH, while autophagy inhibitor aggravated TH loss and α-syn accumulation. aFGF and autophagy agonist alleviated the abnormal expression of protein. These results demonstrated that aFGF can alleviate PD development by regulating autophagy.

In current study, using autophagy inhibitors and agonists, it was found that aFGF can alleviate PD development by regulating autophagy. DAergic death contributes to the development of PD. *In vitro*, TUNEL staining result have further demonstrated that aFGF attenuates apoptosis of PC12 cells, which is consistent with the results that in autophagy agonist group. It has been reported that aFGF promotes autophagy level and consequently contributes to the recovery of spinal cord injury. Conversely, our and others’ studies have shown that autophagy level is enhanced during PD, and some LC3B positive cells colocalize with TUNEL positive signals (Niu et al., 2018), which may suggest that regulatory mechanisms are different in different type of nerve injuries.

6-OHDA-induced PD rats model are considered to be a standard model, and studies have confirmed that 6-OHDA can act as an ER stress inducer (Yamamuro et al., 2006). Therefore, ER stress activators were not added in this study. Previous studies have shown that aFGF protects DAergic by inhibiting ER stress (Wei et al., 2014). Both ER stress and autophagy are involved in the pathological process of PD. But the relationship between them is still unclear in PD. Previous studies have shown ER stress can regulate autophagy (Hetz et al., 2009; Senft and Ronai, 2015). In this study, the ER stress inhibitor was added to investigate the regulation of autophagy by ER stress. The studies *in vivo* and *in vitro* have showed that inhibition of ER stress promoted autophagy. It is indirectly shown that aFGF can protect neurons by inhibiting ER stress to promote autophagy.

Since the neuroprotective effect of aFGF is mainly through binding with FGFR1 (Li, 2019). Specifically, FGFR1 is expressed on TH-positive cells of the SNpc and terminals in striatal (Boshoff et al., 2018). Our study also showed that FGFR1 level was increased after PD (**Figure 2**), while the expression of FGFR1 was similar in the sham group and aFGF treatment group, which may suggest that FGFR1 will transfer into the nucleus due to injury. After administration, it promotes the transfer of FGFR1 to the membrane, thereby promotes the repair of neuronal damage. In contrast, studies have shown that inhibition of the FGFR1 signaling pathway in the pathogenesis of temporomandibular joint osteoarthritis activates autophagy level, thereby ameliorates disease (Wang et al., 2018), probably due to FGFR1 having the different effects on different tissue.

The PD is characterized by degenerative DAergic degeneration, which induces excessive neuronal apoptosis. CHOP is a critical protein that involved in ER stress-induced apoptosis. During prolong ER stress, CHOP expression rapidly increases and leads to apoptosis. TRB3 is an inducing factor associated with neuronal death, and it is associated with ER stress-induced apoptosis (Ohoka et al., 2005). Misfolded protein Aβ induces increases of TRB3 in neurons during PD development (Saleem and Biswas, 2017). It is shown that elevated expression of TRB3 is associated with DAergic death (Aim et al., 2015). Recently, it is unclear the relationship of ER stress and autophagy during aFGF protecting DAergic. Therefore, this study explored the role of TRB3 on this process. The results showed that the 6-OHDA treatement induces TRB3 expression and decreases the expressions of the autophagy-related indicators, mTOR and LC3B/A, which was reversed by aFGF treatment. Previous studies have shown that TRB3 blocks autophagic flow (Saleem and Biswas, 2017), and this study also confirmed that elevated TRB3 also causes increased p62 (an indicator of autophagosome clearance), indicating that autophagic flow is impaired in PD, and TRB3 binding to p62 affects the clearance of autophagosomes (Huang et al., 2015).

Our current study had showed that aFGF and autophagy inducer treatment increased the survival rate of PC12 cells. Moreover, TRB3 was significantly increased in 6-OHDA-induced PD animals and cell models, and aFGF reduced TRB3 expression. Conversely, studies have shown that TRB3 enhances autophagy (Tsai et al., 2018), leading to autophagic death of cells, which may cause excessive expression of TRB3 with varying degrees of stress, thereby aggravating cell death. Moreover, the FGF signaling pathway is involved in the activation of blastocysts by activating autophagy (Shin et al., 2017). These results indicate that aFGF ameliorates 6-OHDA-induced neuronal death *via* down-regulating the pro-apoptotic protein of TRB3 *in vivo* and *in vitro*.

## Conclusion

This study has demonstrated that aFGF administration ameliorates 6-OHDA-induced PD development. Mechanistic study reveals that aFGF activates autophagy level by inhibiting ER stress-induced TRB3 overexpression during PD development, and subsequently ameliorates 6-OHDA-induced neuronal apoptosis. Our current study firstly elucidates the role of autophagy and its relationship with ER stress during aFGF treatment for PD, which may provide a new potential target for clinical treatment of PD.

## Data Availability Statement

All datasets generated for this study are included in the article/supplementary material.

## Ethics Statement

The animal study was reviewed and approved by Laboratory Animal Ethics Committee of Wenzhou Medical University.

## Author Contributions

JY and YW conceived and designed the experiments. XZ, BW, and GZ performed the experiments. XZ, YW, and JiX performed statistical analysis and wrote the paper. YY, XH, JuX, PZ, YL, and KX provided assistance with experiments. All authors discussed the results and approved the final manuscript.

## Conflict of Interest

The authors declare that the research was conducted in the absence of any commercial or financial relationships that could be construed as a potential conflict of interest.
